# Introducing Fiber-Assisted Colorimetric Measurements as a Quality Control Tool of Hot Melt Extruded Filaments

**DOI:** 10.3390/pharmaceutics14051055

**Published:** 2022-05-14

**Authors:** Rebecca Chamberlain, Eirini Mangiorou, Björn Fischer

**Affiliations:** Institute of Pharmaceutics and Biopharmaceutics, Heinrich Heine University, Universitätsstraße 1, 40225 Düsseldorf, Germany; up1055165@upnet.gr (E.M.); bjoern.fischer@hhu.de (B.F.)

**Keywords:** CIELAB measurements, colorimetry, pharmaceutical filaments, color-coded filaments, hot melt extrusion, analytics of extruded filaments, personalized medicine, fixed-dose combinations, FDM printing, quality control

## Abstract

Pharmaceutical and medicinal printing technologies allow personalization and on-demand manufacturing of drug and medicinal products. Being able to manufacture patient tailored dosage forms or medical devices in a pharmacy, medical office, dental laboratory, or hospital at the point of care raises new demands on quality control procedures. For Fused Deposition Modeling, for example, it must be ensured that the starting materials, the (drug-loaded) filaments, are not accidentally exchanged by the operator. This study investigated the potential of colorimetric measurements for direct and indirect determination of the identity of extruded filaments consisting of polymer matrix, different API and/or coloring agents. Since reflection measurements were affected by surface properties of the filaments, a self-constructed filament holder was utilized with an optical fiber positioned in a 180° angle to a white light LED to perform transmission measurements. It was possible to distinguish filaments with different API concentrations by their color values, taking into account that transmission partially decreased by increased API concentration. Therefore, the intensity of the light source had to be adjusted depending on the transparency of the filament. It was shown that colorimetry can be used as a quality control tool to detect differences in drug-loading and is able to distinguish various extruded batches. Additionally, if differences in API/polymer concentrations do not lead to significant changes in L*a*b values, coloring agents were used as additives in low concentrations to color code filaments. In future studies, the setup must be supplemented with a standardized light source and obscuring filters for light intensity adjustments.

## 1. Introduction

**Figure 1:**
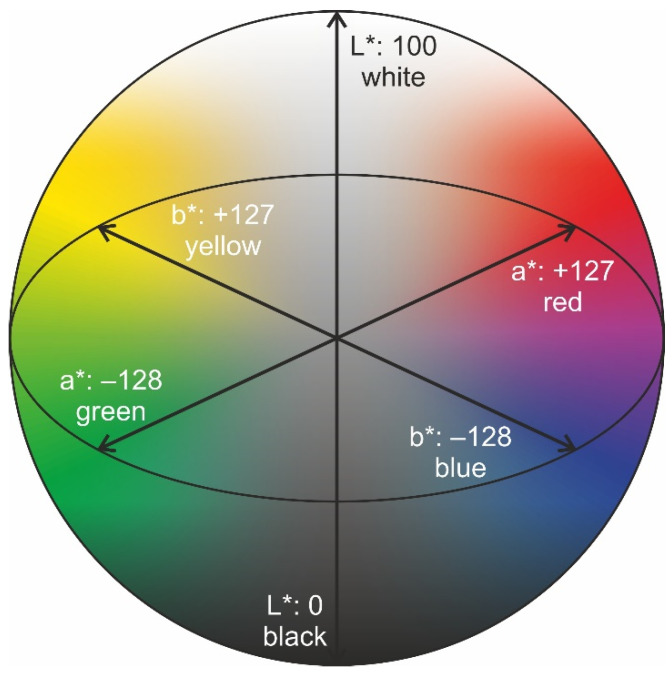
L*a*b* three-dimensional color space.

The application of 3D printing for patient-specific therapy is gaining more and more interest, as medical and pharmaceutical 3D printers as well as medical grade printing materials are already commercially available. The “one-size-fits-all” approach is being replaced more and more by targeted and personalized medicine [1]. With the help of medical Fused Deposition Modeling (FDM) 3D printing, patient-specific medical devices, such as implants, prostheses, and surgical guides are printed using biocompatible materials (e.g., PCL, PLA, PVA). Additionally, non-biocompatible materials (e.g., ABS, TPU) are used for printing medical models for perioperative surgical planning and simulations [2]. In pharmaceutical FDM 3D printing, various thermoplastic polymers are processed by hot melt extrusion (HME) with active substances [3]. The intermediate filament is then used to produce solid dosage forms such as tablets, capsules, inserts, implants, and films [4]. The ongoing research may lead to a future where solid dosage forms will be printed at the point of care (PoC) allowing personalization of drug content, drug release, and the supply of medicines containing more than one drug for the treated patient [5]. To avoid incorrect application when using a wide variety of pharmaceutical grade filaments, for example in hospitals or local pharmacies, the fact that the matrix, the active ingredient, and/or other additives are responsible for the coloration of the filaments might be used for colorimetric measurements. In this context, the CIE L*a*b* (CIELAB) color space introduced by the International Commission on Illumination in 1976 was utilized, where colors are defined by three unique values: L* for lightness, a* for red and green color, and b* for blue and yellow color [6]. While L*-values can vary between 0 (black) and 100 (white), both a* and b* values range from −128 (a*: green, b*: blue) to +127 (a*: red, b*: yellow), which leaves theoretically 6.5 million possibilities for describing different colors [7]. These L*a*b* values are distributed in the three-dimensional space in such a way that a sphere results (Figure 1). For pharmaceutical applications, colorimetry has already been tested as a quality control (QC) tool for various drug preparations since it represents a non-destructive characterization method [8]. For tablet analysis, Siddiqui et al. could determine a correlation between the surface color and tensile strength of tablets and were also able to detect deviations in tablet hardness through differences in color measurements [9]. Differences in surface color of coated tablets were studied by Bogdansky [10]. Berberich et al. were able to determine the period of time uncoated tablets remained white during storage by color measurement [11]. Gren and Nyström used colorimetry for an estimation of the surface coverage of particles with colored and uncolored stearic acid [12]. Barimani et al. used CIELAB measurements for visual inspection of tablet coating produced with continuous direct compression to ensure a defect-free coating of each measured tablet [13]. CIELAB measurements were also performed by Lakio et al., who analyzed granules and pellets as color indicators for potential heat stress during drying processes [7]. Wickström et al. detected vitamin B containing layers conducted by Inkjet-Printing via differences in CIELAB values but were limited to differentiating between the first 5–6 printed layers since the b* value reached color saturation [14]. The findings of the listed studies were taken as an example to use the distinguished extruded pharmaceutical filaments. For this purpose, a filament holder was designed, where the filament could be inserted and both reflection and transmission measurements could be performed. An optical multi-core fiber was used, which can both transmit light from a white light LED towards the sample and collect light after reflection. The same fiber was used collecting light from a second LED after being transmitted through or partly directed around the filament. The fade of the applied electromagnetic radiation and light scattering effects caused by the filament geometry and composition are not analyzed in depth in this study. In this study, the aim was to investigate, whether the difference in concentration of an active pharmaceutical ingredient (API) is reflected in measurable color difference of the filaments. Pramipexol (P), levodopa (LD), benserazide (BZ), and praziquantel (PZQ) served as model APIs. Since the active ingredients led to a change in transparency, it was important to ensure that the light intensity was appropriate to visualize differences between the active ingredients or active ingredient concentrations. If a dose increase and associated change in polymer-API concentration did not result in a desired color change, a pharmaceutical coloring agent was added to the formulation [15,16,17,18]. Here, it was necessary to conduct preliminary tests without an active ingredient to determine which differences in concentrations of the additive led to significantly different color values in the CIELAB color space. The added color agents were either dissolved or dispersed in the polymer melt. The bright yellow, crystalline powder riboflavin (RF), also known as vitamin B2, and indigo carmine (IC), a blue coloring agent, were chosen to serve as additives. Thus, when both coloring agents were used (yellow and blue) the extruded filaments appeared green. Thereby, only slight differences in the concentration of the coloring agent were chosen to analyze whether these were also reflected in different color values. The dosing accuracy during extrusion was examined in content uniformity studies conducted via high-performance liquid chromatography (HPLC) analysis. These experiments were performed to develop new analytical techniques for dosage investigation, batches of filaments consisting of different concentrations of API, and indirect determination using a coloring agent on the polymer-API composition using CIELAB measurements.

## 2. Materials and Methods

The polymers including ethylene-vinyl acetate copolymer (EVA, 82:18, Escorene^®^, FL 01418, TER Chemicals, Hamburg, Germany) and vinylpyrrolidone-vinyl acetate copolymer (VA, 6:4, Kollidon^®^ VA 64, BASF, Ludwigshafen am Rhein, Germany) served as polymer matrices. Four thermal stable model substances, praziquantel (PZQ, Bayer AG, Leverkusen, Germany), levodopa (LD, Zhejiang Wild Wind Pharmaceutical Co. Ltd., Dongyang, China), benserazide hydrochloride (BZ, s.p. Quimica, Tarragona, Spain), and pramipexole dihydrochloride monohydrate (P, Chr. Olesen, Copenhagen, Denmark) were tested in this study. For coloring agents, riboflavin (RF, Caesar & Loretz GmbH, Hilden, Germany) and sodium 3,3′-dioxo-[2,2′-biindolinylidene]-5,5′-disulfonate (indigo carmine, IC, bld pharmatech GmbH, Kaiserslautern, Germany) were used.

### 2.1. Hot Melt Extrusion

All filaments were prepared by HME using a co-rotating twin-screw extruder with a hot-melt extrusion die (Pharmalab HME 16, Thermo Fisher Scientific, Waltham, MA, USA). A gravimetric feeder (K-SFS-24/6, Coperion, Stuttgart, Germany) was used for all experiments. An in-house manufactured die with a diameter of 1.85 mm was used. The desired filament diameter was achieved using a belt hauled-off unit of a winder (Brabender, Duisburg, Germany) with a belt speed of 1.5–1.9 m/min and the filament was pulled through a roll-system with four 360° air flow ring nozzles (Super Air Wipe, Exair, Cincinnati, OH, USA) for active cooling of the filament. With a laser-based diameter measurement module (Laser 2025 T, Sikora, Bremen, Germany) the filament diameter was controlled [19]. The screw speed was set to 30 rpm and powder feed rate to 5 g/min. The screw configuration included two kneading zones and a degassing port was installed [20]. All temperatures of the heating zones were set for each extrusion according to Table 1 depending on the polymer matrix of the filament [21,22]. The powder mixtures were blended for 20 min in a Turbula^®^ mixer. Filament samples were only collected when extrusion runs reached equilibrium condition for drug content [20].

### 2.2. Colorimetry Measurements of Filaments

The hot melt extruded filaments were cut into filament sticks of 4 cm and were placed in an in-house produced stainless steel sample holder with an inner diameter of 1.9 mm. Dual output channels of the power supply (Rigol DP832, Meilhaus Electronic GmbH, Alling, Germany) were utilized for the regulation of two light-emitting diodes (LED, 333-2UTC/S400-A6, Everlight, Hsinchu, Taiwan). For the assembly of the individual components for colorimetric measurements, a system bar, sliders, a mounting cube, a tower, and a height adjustable table were obtained from OWIS^®^ (OWIS GmbH, Staufen im Breisgau, Germany).

**Figure 2:**
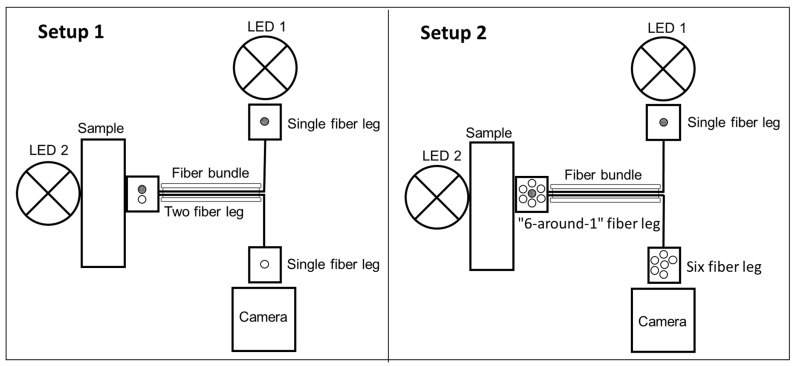
Assembly of the components for reflection and transmission measurements for Setup 1 and 2: light is applied from LED 1 and enters and exits at the same fiber bundle or light from LED 2 passes through the filament and transmitted light is collected into the fiber bundles. A picture of the fiber leg (single- or six-fiber leg) was taken by a camera.

For reflection measurements, a dual fiber optical probe with SMA connectors (SMA905, Ø600 µm, 300–1200 nm, BFY600HS02, Thorlabs, Newton, NJ, USA) was attached to the sample holder and was guided from the LED to the sample and backwards to the camera (WIFI Digital Microscope 8595776342, AHSNOPTIC, Beijing, China). In a second setup (Setup 2, Figure 2), the dual fiber bundle was exchanged to a multi-core fiber probe (RP21, Ø200 µm, 400–2400 nm, FG200LEA, Thorlabs, Newton, NJ, USA), whereby a single fiber leg was directed to the first LED for reflection measurements. The probe leg “6-around-1” fiber configuration was guided to the sample and the 6-fiber leg was directed to the camera. For transmission measurements, a second LED was installed in an 180° angle to the above-mentioned fiber probes using the same orientation of the fiber legs and the same camera (Figure 2). The fibers were directly attached to the filament holder or fixed on adjustable sliders (OWIS GmbH, Staufen im Breisgau, Deutschland) in front of the LED or the camera with SMA connectors. Through an integrated metal platform tensioned by two springs within the holder, the filament was always in contact with the curvature facing the fiber optic probe to ensure accurate positioning.

### 2.3. Data Evaluation of Colorimetry Measurements

**Figure 3:**
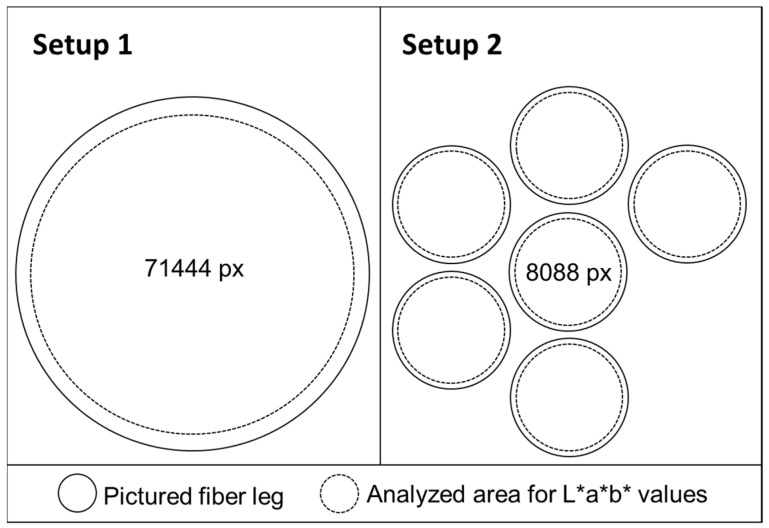
Schematic visualization of the pictured fiber leg for Setup 1 and 2 with the selected area to be analyzed (for Setup 1: 71444 Pixel; and for Setup 2: six fiber legs with each 8088 Pixel) for one filament in Corel PHOTO-PAINT.

The mean L*, a*, b* values of the pictures illustrating the respective fiber leg were obtained by analyzing a defined area via Corel^®^ PHOTO-PAINT X7 (17.6.0.1021). Mimicking the circular surface of the fiber, a circular area was selected as the to be analyzed area, but edge areas were left out (Figure 3). Only one L*, a*, and b* value per filament was measured in the first set up, since a single fiber leg was analyzed. For the second set up, six circular areas were evaluated separately or a mean value of all six fiber bundles with standard deviation was calculated for each filament.

### 2.4. Reference Method for Determination of Drug Content (HPLC-UV)

For sample preparation, filaments were dissolved in flasks (*n* = 10), which were filled with demineralized water up to 50 or 100 mL. All the agents that were used except EVA are water soluble. The content of drug-loaded filaments was determined by high performance liquid chromatography (HPLC) analysis. The HPLC system (Dionex, Sunnyvale, CA, USA) was equipped with a quaternary pump (P 580 A, Dionex, Sunnyvale, CA, USA) and an autosampler (ASI-100, Dionex, Sunnyvale, CA, USA). A 250 × 4.6 mm column (Eurospher II 100-5 C18A, Knauer, Berlin, Germany) with an integrated precolumn was used. Three different HPLC methods were used for the analysis of the active and color ingredients. For the first method, ammonium acetate buffer and methanol (mobile phase B) served as eluents. The gradient was set as follows: mobile phase B was increased from 1 to 5% (*v/v*), within the first minute, held at 5% (*v/v*) for 4 min, increased from 5 to 10% (*v/v*) within 1 min, held at 10% (*v/v*) for 4 min, increased again from 10 to 20% (*v/v*) within 1 min, held for 4 min at 20% (*v/v*), increased again from 20 to 99% (*v/v*) within 5 min, held for 2 min at 99% (*v/v*) and decreased to 1% (*v/v*) within 0.5 min, again until 22.5 min after sample injection [23]. An equilibration time of 3.5 min per run was maintained before the next sample was injected. An injection volume of 200 µL was chosen to analyze benserazide (280 nm), levodopa (280 nm), pramipexole (264 nm), and indigo carmine (280 nm). The second HPLC method was used to quantify praziquantel at the wavelength of 280 nm. Phosphate buffer and acetonitrile (mobile phase B) served as eluents and the flow rate was 1.5 mL/min. The gradient was set as follows: mobile phase B was increased from 30 to 95% (*v/v*), within the first 8 min, held at 95% (*v/v*) for 3 min, decreased to 30% (*v/v*) within 1 min, again until 12 min after sample injection. An equilibration time of 2 min per run was kept before the next sample was injected. The third HPLC method was necessary to evaluate the concentration of riboflavin (280 nm). Phosphate buffer and acetonitrile (mobile phase B) served as eluents and the flow rate was 1.0 mL/min. The gradient was set as follows: mobile phase B was stable at 10% (*v/v*) the first 5 min, increased to 20% (*v/v*), the next 15 min, held at 20% (*v/v*) for 5 min, increased again to 50% (*v/v*) within 10 min, held at 50% (*v/v*) for another 10 min, and decreased to 10% (*v/v*) within 2 min, again until 47 min after sample injection [24].

## 3. Results

### 3.1. Transmission and Reflection Measurements of Filaments

**Figure 4:**
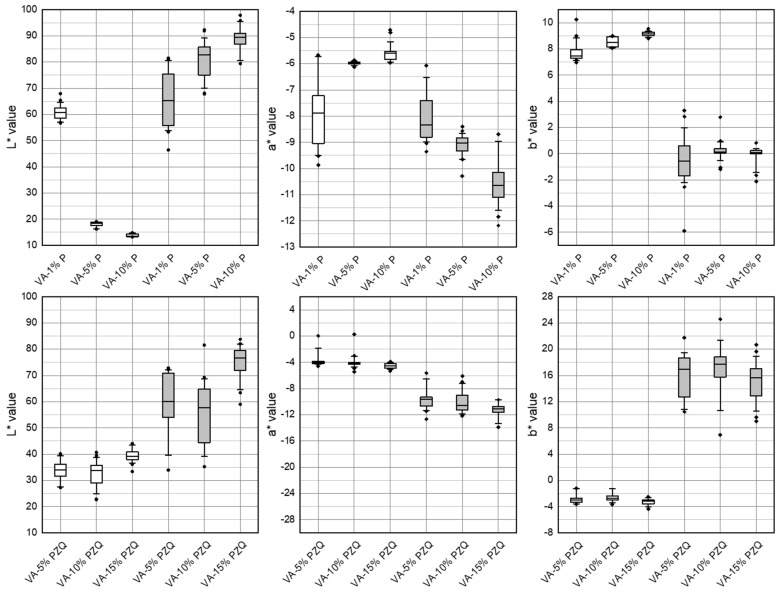
Box-whisker plots of L*a*b* values of transmission (white) and reflection (grey) measurement of VA-1,5,10% P and VA-5,10,15% PZQ filaments obtained with Setup 1 (*n* = 10, Q1/Q3 ± 1.5 IQR, outliers).

When comparing reflection and transmission measurements, it is noticeable that the resulting L*a*b* values of the two measurement techniques using Setup 1 (Figure 2) give very different results when analyzing the same filament batch. For example, the L* value obtained by a transmission measurement of filaments consisting of vinylpyrrolidone-vinyl acetate copolymer and 5% (*w/w*) pramipexole (VA-5% P) is 18.4, and reflection measurement for the same batch the median L* value is 82.6 (Table 2). All b* values obtained by transmission measurements differ from the results of the reflection measurements of both the three batches with pramipexole and the three batches with praziquantel. While the median b* value obtained by the transmission measurement of VA-1% P is 7.4, of VA-5% P is 8.5, and of VA-10% is 9.1, the median b* values obtained by reflection measurements for all three batches are −0.2 ± 0.3. b* values of −3.0 ± 0.3 were measured for all three batches of vinylpyrrolidone-vinyl acetate copolymer-praziquantel filaments with a drug load of 5, 10, and 15% (*w/w*) (VA-5% PZQ, VA-10% PZQ, VA-15% PZQ) using the transmission assembly of Setup 1. Reflection measurements of these praziquantel filaments obtained median b* values of 16.7 ± 1.1. Similar values for transmission and reflection measurements were found for the L* values of VA-1% P filaments (transmission: $\bar{x}\;$ = 60.4, reflection: $\bar{x}\;$ = 66.7) and for the a* values of this batch (transmission: $\bar{x}\;$ = −7.9, reflection: $\bar{x}\;$ = −8.3). Nevertheless, it can be concluded that when specifying the L*a*b* values, it should always be mentioned at which angle the emitted light is collected by the fiber and, therefore, specify if the filament was analyzed in reflection or transmission. The reflectance measurements show higher deviations of L*a*b* values compared to the transmission measurements (Figure 4). The determination of the filament content does not reflect the large deviations of the reflection measurements, so it was concluded that this was due to the measurement method. The high variations might be caused by the varying positioning of the filaments to the two-fiber leg due to the cylindrical geometry of the sample, by composition of the matrix (e.g., suspended particles) and by surface irregularities resulting in complex scattered light effects. Thus, it was decided that only transmission measurements would be performed for further measurements. However, it is noticeable that the L*a*b* values of the transmission measurements for all three batches of VA-PZQ filaments show very similar values. For VA-P filaments, on the other hand, a different L* value occurs for the filament batch of VA-1% P compared to the other two filament batches with pramipexole.

This can be explained since pramipexole is suspended in the VA matrix and filaments with 1% pramipexole are transparent and both the 5% and the 10% pramipexole filaments are not translucent. Consequently, the VA-1% P filament batch can be clearly distinguished from the VA-5% P and VA-10% P batches by the L* value, although the b* value is very similar compared to the other two pramipexole batches and the a* value shows a very high deviation and does not show a significant difference. To further optimize the transmission measurements and reduce the potential uncertainty of differences in reflection of the incident light caused by the curvature of the filaments, a new fiber bundle with a 6-fiber end was used (Setup 2, Figure 2).

### 3.2. Optimization of Transmission Measurements with Setup 2 and Voltage Adaption

Compared to Setup 1, the emitted light was not collected by only one fiber, but by six circular arranged fibers (“6 around 1” fiber leg) so that light was captured from six different angles. In the 6-fiber leg, the fibers are arranged in such a way that one fiber is centered, which can lead to irritations since the fiber was circularly arranged at the sample side. It is noticeable that the captured images of the 6-fiber leg show different L*a*b* values for the six individual fibers (Table 3).

A regularity could not be found with respect to which fiber was consistently darker or lighter (L* value), or whether the a* and b* values showed systematic tendencies. The L* value shows the greatest fluctuations for the VA-1% P filament and varies between 49.8 (fiber 1) and 58.9 (fiber 6). Here, the b* values also differ by 5.0 (9.0 and 4.0), but the a* values are nearly the same (−8.6 and −8.1). For the other measured values, the fluctuations of the L*a*b* values are in the range of 0.5–1.8. However, the obtained data lead to the assumption that there was a variability in L*a*b* values due to the geometry of the filaments. Additionally, the influence of the brightness of the imaged fiber on the a* and b* values was investigated by varying the light intensity of the LED. The different L*a*b* values of the six fibers of EC- 20% LD/5% BZ, VA-5% P, and VA-10% PZQ filaments by varying the voltage of the LED from 2.30 to 3.20 V are listed in Table 4.

**Figure 5:**
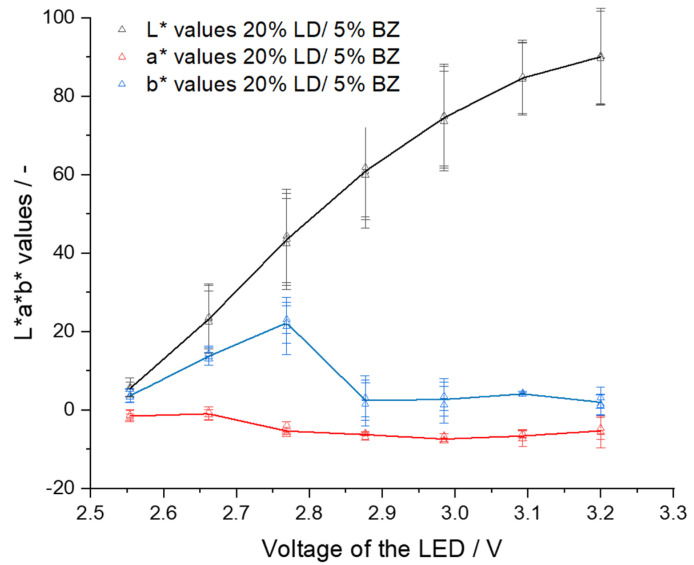
L*a*b* values depending on LED voltage for EC- 20% LD/ 5% BZ filaments (mean ± SD, three filaments, *n* = 6).

**Figure 6:**
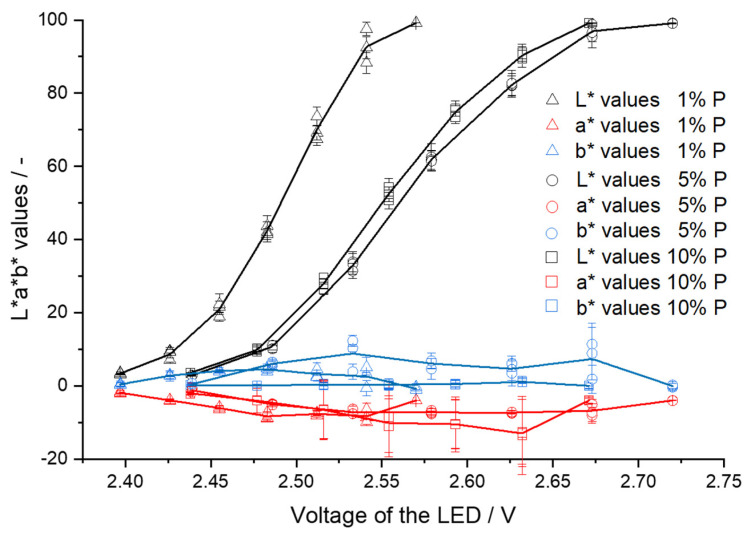
L*a*b* values depending on LED voltage for VA-1% P, VA-5% P, and VA-10% P filaments (mean ± SD, each of the three filaments, *n* = 6).

**Figure 7:**
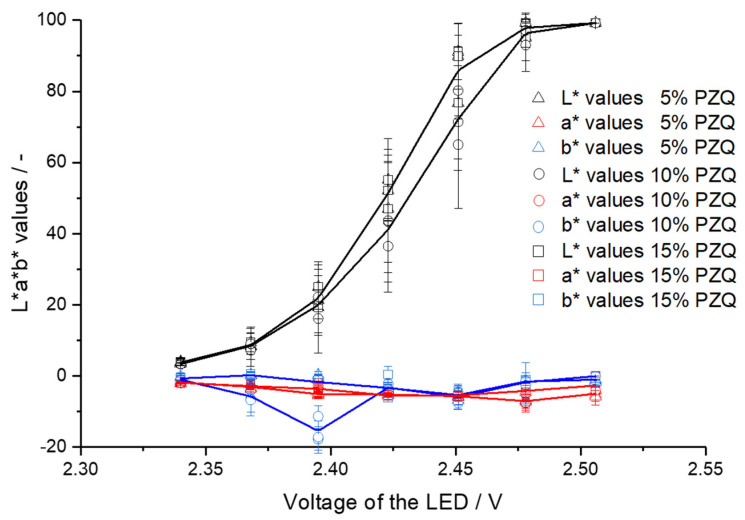
L*a*b* values depending on LED voltage for VA-5% PZQ, VA-10% PZQ, and VA-15% PZQ filaments (mean ± SD, each of the three filaments, *n* = 6).

By setting low volt values, the fibers appear black, and at high volt values, they appear white. In both cases, color differences can no longer be observed between the six fibers. If the filaments were not transparent and/or colored (EC- 20% LD/ 5% BZ filaments) a higher voltage of the LED had to be set so that color values of the respective fibers could be determined. For EC- 20% LD/ 5% BZ filaments 2.80 V were applied to ensure that the brown color of the filaments could be determined from the fibers. For VA-5% P filaments, voltages below 2.40 led to black fibers and voltages above 2.80 V resulted in white fibers. Similarly, for VA-10% PZQ filaments voltages above 2.80 V led to white fibers. Since the filaments were transparent, 2.50 V still caused fibers to appear white, but at 2.40 V the fibers were already very dark. To understand the light intensity dependent influence on a* and b* values, the voltages were systematically changed and the L*a*b* values were determined (Figure 5, Figure 6 and Figure 7). There was a noticeable difference between the a* and b* values: the brighter or darker the imaged fiber was, the closer the two values approached to zero. This can be explained by the fact that the L*a*b* color space is represented three-dimensionally as a sphere (Figure 1). White has the L*a*b* value of L* 100 a* 0 and b* 0 and black has the L*a*b* value of L* 0 a* 0 and b* 0 and consequently the color value of a very bright fiber approaches the L*a*b* values of white and dark fibers approach the L*a*b* values of black [25]. We also observed that the L*a*b* values for the filaments changed as a function of the brightness of the LED, respectively, the voltage setting. For EC- 20% LD/ 5% BZ filaments, throughout the range of L*, the a* values only slightly decrease. However, the b* value indicates a maximum value of 22.0 ± 7.3 at 2.77 V, when the L* value is 43.4 ± 11.8 (Figure 5). This is consistent with the observations in Table 4, where the setting of 2.80 V shows fibers that appear yellow/brown, which affects the b* value.

For the three filament batches with different pramipexole concentrations, the a* and b* values differ regarding the voltage of the LED (Figure 6). For VA-1% P filaments, the highest b* value of 6.06 is at 2.47 V, for VA-5% P filaments the highest b* value is at 2.63 V and corresponds to 9.61, and for VA-10% P filaments the highest b* value of 8.84 was measured at 2.52 V. The range for VA-1% P filaments, in which the measured a* and b* values differ from −4 ± 6, is from 2.40 to 2.57 V.

**Figure 8:**
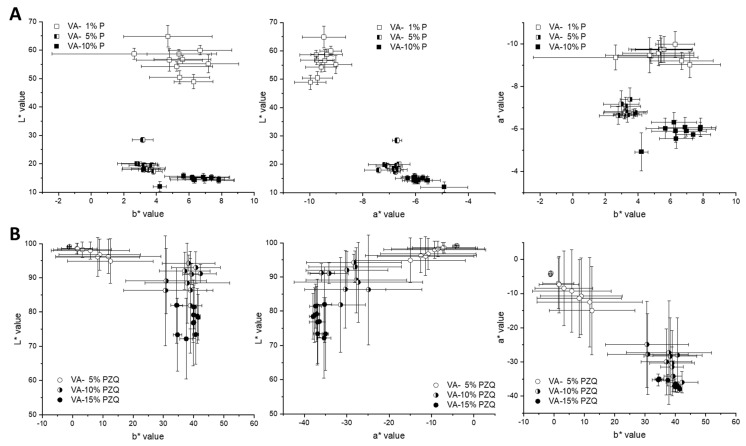
The 2D graphs of L*a*, L*b*, and a*b* values at 2.50 V for (**A**) pramipexole filaments (1, 5, 10% (*w/w*)) and (**B**) praziquantel filaments (5, 10, 15% (*w/w*)).

For the other two pramipexole batches, the range is between 2.44 and 2.67 V (VA-5% P filaments) and between 2.44 and 2.72 V (VA-10% P filaments). For the comparative measurements of the pramipexole batches, 2.50 V was selected, which was in the mentioned detection ranges and at the same time resulted in differences in L*a*b* values of the three batches. The same measurements were performed with all three praziquantel filament batches (Figure 7). The a* and b* values of all three batches varied from −8 to 1 despite one outlier at −17 (b* value of VA-10% PZQ batch), which indicates the difficulties in seeing significant differences between the three batches with different API contents. For VA-5% PZQ filaments, the minimum b* value of −5.69 is at 2.42 V, for VA-5% PZQ filaments the lowest b* value is at 2.43 V and corresponds to −5.66, and for VA-10% PZQ filaments the lowest b* value of −5.89 was measured at 2.44 V. The range for VA-5% PZQ filaments, in which a* and b* values differ from 0 ± 1, is from 2.39 to 2.48 V. For the other two praziquantel batches, the range is between 2.41 and 2.45 V (VA-10% PZQ filaments) and between 2.36 and 2.47 V (VA-15% PZQ filaments). Again, 2.50 V was selected for the comparative measurements of the three praziquantel batches. Distinct clusters are noticeable in the 2D graphs of the pramipexole batches (Figure 8A). Thus, the VA-1% P filaments are characterized by the highest L* values and the lowest a* values. The b* values are similar to the b* values of the other two batches. The clusters of VA-5% P filaments and VA-10% P filaments are closer to each other. However, the L* values of VA-5% P filaments are higher and both the a* and b* values are lower in comparison to the a* and b* values of VA-10% P filaments. Clusters can also be identified for the praziquantel-containing filaments (Figure 8B). However, it is not possible to see significant differences between the batches since the standard deviations are very high and overlap for all three values. A trend can be recognized that with increasing concentration of praziquantel the L* and a* decrease, and the b* value increases. As the three different praziquantel batches could not be significantly distinguished by colorimetric measurements, two coloring agents, which are commonly used in pharmaceutical coating processes [26,27], were added in different concentrations to create variability in L*a*b* values due to additives.

### 3.3. Color-Coded Filaments

To be able to distinguish filament batches by colorimetry, as the polymer-API filament compositions do not lead to a desired difference, coloring agents commonly used in pharmaceutical production were added to the powder mixture and were incorporated together with VA and praziquantel by HME. The excipients riboflavin (yellow) and indigo carmine (blue) caused the filaments to appear green. By maintaining the riboflavin (RF) concentration and only increasing the indigo carmine (IC) concentration, the ratio of additives changed from 1:1 (0.1% (*w/w*) RF, 0.1% (*w/w*) IC) for VA-5% PZQ filaments to 1:3 (0.1% (*w/w*) RF, 0.3% (*w/w*) IC) for VA-10% PZQ filaments, and to 1:5 (0.1% (*w/w*) RF, 0.5% (*w/w*) IC) for VA-15% PZQ filaments. The concentrations of the three ingredients (API and coloring agents) were analyzed by HPLC analysis and are shown in Table 5.

**Figure 9:**
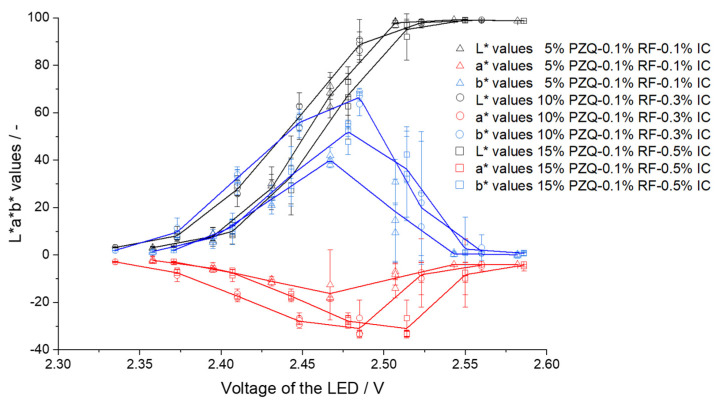
a* and b* values depending on LED voltage for the filaments of PZQ with different concentrations of RF and IC (VA-5% PZQ-0.1% RF-0.1% IC, VA-10% PZQ-0.1% RF-0.3% IC and VA-15% PZQ-0.1% RF-0.5% IC).

**Figure 10:**
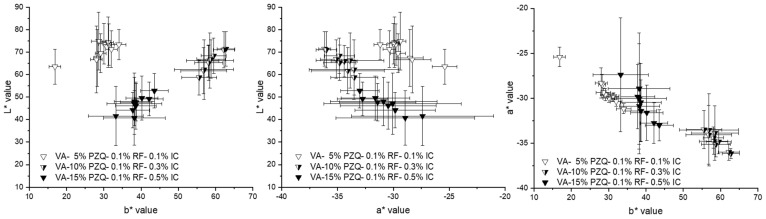
The 2D graphs of L*a*, L*b*, and a*b* values at 2.50 V for color-coded praziquantel filaments (VA-5% PZQ-0.1% RF-0.1% IC, VA-10% PZQ-0.1% RF-0.3% IC and VA-15% PZQ-0.1% RF-0.5% IC).

Again, seven different voltages were tested, and 2.50 V was selected for the comparative measurements of the three green praziquantel batches (Figure 9). According to the expectations, a significant difference in the L*b* 2D graph was found due to the addition of excipients that affect the b* value significantly. Why the b* values of VA-15% PZQ-0.1% RF-0.5% IC filaments are lower than those of A-10% PZQ-0.1% RF-0.3% IC cannot be explained by the higher amount of indigo carmine, but suspended particles must have an influence here. HPLC analysis showed that the concentration of IC increases with increasing concentration of praziquantel (Table 5). It was not investigated whether IC was dissolved or suspended in the polymer matrix and the saturation concentration of IC in VA was not determined. But blue particles were visible within the filament indicating suspended IC particles. However, by the addition of the two coloring agents to the formulation, the individual produced batches differ significantly from each other in the L*b* 2D graph (Figure 10).

## 4. Conclusions

When performing colorimetric measurements with pharmaceutical filaments, this method can be used for distinguishing extruded filament batches with different API concentrations. Using a setup optimized for pharmaceutical filaments, extruded batches could be distinguished despite color differences generated by the amount of API or after using colored additives. The filaments were analyzed by a six-fiber bundle to depict light scattering effects caused by the filament geometry with the consequence that the fibers show high deviations. Despite the high deviations, clusters can be found in 2D graphs of L*a*, L*b*, and/or a*b* values which indicate a significant difference in the individual batches. If the matrix and the desired API concentration does not lead to differences in color changes despite other filaments, adding other excipients is a reasonable approach to achieve clustering of L*a*, L*b*, and/or a*b* values. Ensuring that the filaments were optimally illuminated, the power supply of the LED was regulated so that the a* and b* values of the analyzed filaments were not zero (black or white) because the fibers were either too dark or too bright. To further optimize the setup, measurements should be performed with a standardized light source at the same voltage and darkening needs be realized by additional optical filters. By implementing a filament holder, colorimetric measurements might also be performed during the HME process. Since the HME process is also used to manufacture granules and amorphous solid dispersions, this tool could be adapted to these (intermediate) drug products. The fiber-assisted colorimetric analysis with the small-sized components can be easily installed after a few modifications in the downstream equipment of the HME process.

## Figures and Tables

**Table 1:** Temperature profile of zones 2–10 for extrusion runs with selected polymers.

Polymer	Temperature Profile in Zone 2–10/°C								
	2	3	4	5	6	7	8	9	10
EVA	20	20	50	85	85	85	85	85	85
VA	20	30	75	165	180	180	180	180	180

**Table 2:** L*a*b* values of transmission and reflection measurement of the filaments obtained with Setup 1 (*n* = 10; mean, Q1-Q3) despite the analyzed content uniformity determined by HPLC analysis (*n* = 10; mean, SD).

	Transmission						Reflection						Drug Content/%	
	L* Value		a* Value		b* Value		L* Value		a* Value		b* Value			
Batch VA-	$\bar{x}$	Q1–Q3	$\bar{x}$	Q1–Q3	$\bar{x}$	Q1–Q3	$\bar{x}$	Q1–Q3	$\bar{x}$	Q1–Q3	$\bar{x}$	Q1–Q3	$\bar{x}$	SD
1% P	60.3	4.5	−7.9	1.9	7.4	0.6	66.7	19.9	−8.3	1.5	−0.5	2.3	0.98	0.15
5% P	18.4	1.3	−5.9	0.1	8.5	0.8	82.6	11.4	−9.0	0.5	0.1	0.3	5.01	0.13
10% P	13.4	1.2	−5.6	0.3	9.1	0.3	88.3	7.5	−10.6	1.0	0.1	0.2	10.02	0.09
5% PZQ	32.7	6.6	−4.0	0.4	−3.0	0.7	60.1	17.3	−9.6	1.5	17.0	6.3	4.98	0.11
10% PZQ	33.8	8.0	−4.1	0.3	−2.7	0.7	57.0	20.5	−10.5	2.3	17.8	3.5	10.00	0.12
15% PZQ	39.1	3.1	−4.5	0.7	−3.2	0.7	76.6	8.5	−11.1	0.9	15.6	4.3	15.01	0.09

**Table 3:** L*a*b* values determined for fibers 1–6 using Setup 2 of VA-1% P, VA-5% P, and VA-10% P filaments at 2.50 V.

Setup 2	Filament	Fibers		L*, a*, b* Values of Fibers 1–6					
				**1**	**2**	**3**	**4**	**5**	**6**
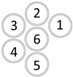	VA-1% P	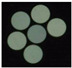	L*	58.9	57.6	53.6	56.6	55.1	49.8
			a*	−8.6	−8.7	−9.0	−9.1	−10.2	−8.1
			b*	9.0	8.6	6.1	8.3	7.4	4.0
	VA-5% P	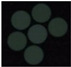	L*	19.7	19.3	20.6	19.4	20.4	21.3
			a*	−7.0	−6.8	−6.7	−6.9	−6.8	−5.9
			b*	3.7	4.2	4.2	4.1	4.2	3.9
	VA-10% P	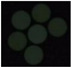	L*	13.3	13.2	16.0	13.2	15.0	16.9
			a*	−6.0	−5.7	−7.0	−7.7	−7.1	−7.0
			b*	3.7	4.2	4.2	4.2	4.2	3.9

**Table 4:** Color observation of transmitted light on selected drug loaded filaments.

Batch	Pictures of Filaments	Voltage of LED with Corresponding Image of the 6-Fiber Leg				
EC- 20% LD/ 5% BZ	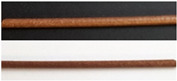	2.30 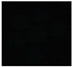	2.40 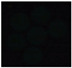	2.50 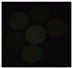	2.80 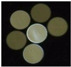	3.20 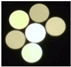
VA- 5% P	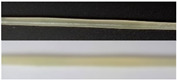	2.30 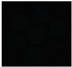	2.40 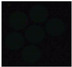	2.50 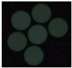	2.80 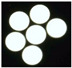	3.20 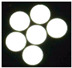
VA- 10% PZQ	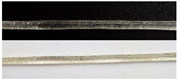	2.30 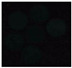	2.40 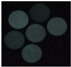	2.50 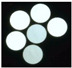	2.80 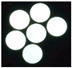	3.20 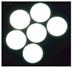

**Table 5:** Content of coloring agents determined by HPLC analysis.

Batch VA-	PZQ Content/%		RF Content/%		IC Content/%	
	$\bar{x}$	SD	$\bar{x}$	SD	$\bar{x}$	SD
5% PZQ-0.1% RF-0.1% IC	4.99	0.10	0.099	0.012	0.102	0.012
10% PZQ-0.1% RF-0.3% IC	10.02	0.09	0.098	0.011	0.299	0.009
15% PZQ-0.1% RF-0.5% IC	15.02	0.12	0.099	0.011	0.500	0.009

## Data Availability

The data presented in this study are available upon request from the corresponding author.

## Abbreviations

3D	three dimensional
ABS	acrylonitrile butadiene styrene
API	active pharmaceutical ingredient
CIELAB	commission internationale de l’éclairage L*a*b*
BZ	benserazide hydrochlorid
EC	ethylcellulose
EVA	ethylene-vinyl acetate copolymer
FDM	fused deposition modeling
HME	hot melt extrusion
HPLC	high-performance liquid chromatography
IC	indigo carmine
LD	levodopa
P	pramipexole dihydrochloride monohydrate
PCL	polycaprolactone
PCL-PVAc-PEG	polyvinyl caprolactam-polyvinyl acetate-polyethylene glycol graft copolymer
PLA	polylactic acid
PoC	point of care
PVA	polyvinyl alcohol
PZQ	praziquantel
QC	quality control
RF	riboflavin
SMA	subminiature version A
T_g_	glass transition temperature
TPU	thermoplastic polyurethane
VA	vinylpyrrolidone-vinyl acetate copolymer
